# DNA Transactions in Bacteria and Membranes: A Place for the Hfq Protein?

**DOI:** 10.3390/membranes15040103

**Published:** 2025-04-01

**Authors:** Sylwia Bloch, Richard R. Sinden, Frank Wien, Grzegorz Węgrzyn, Véronique Arluison

**Affiliations:** 1 Department of Molecular Biology, University of Gdansk, Wita Stwosza 59, 80-308 Gdansk, Poland; sylwia.bloch@ug.edu.pl; 2 Department of Chemistry, Biology and Health Sciences, South Dakota School of Mines and Technology, Rapid City, SD 57701, USA; richard.sinden@sdsmt.edu; 3 Synchrotron SOLEIL, L’Orme des Merisiers, Départementale 128, 91190 Saint Aubin, France; frank.wien@synchrotron-soleil.fr; 4 Laboratoire Léon Brillouin, UMR 12 CEA/CNRS, Bâtiment 563, Site de Saclay, 91191 Gif-sur-Yvette, France; 5 Université Paris Cité, UFR SDV, 35 Rue Hélène Brion, 75013 Paris, France

**Keywords:** Hfq protein, biological membranes, plasmids, transposons, DNA polymerase, outer membrane vesicle cargo, cytoskeletal protein, membrane transertion

## Abstract

DNA metabolism consists of crucial processes occurring in all living cells. These processes include various transactions, such as DNA replication, genetic recombination, transposition, mutagenesis, and DNA repair. While it was initially assumed that these processes might occur in the cytoplasm of prokaryotic cells, subsequent reports indicated the importance of the cell membrane in various DNA transactions. Furthermore, newly identified factors play significant roles in regulating DNA-related cellular processes. One such factor is the Hfq protein, originally discovered as an RNA chaperone but later shown to be involved in several molecular mechanisms. These include DNA transactions and interaction with the cell membrane. Recent studies have suggested that Hfq plays a role in the regulation of DNA replication, mutagenesis, and recombination. In this narrative review, we will focus on the importance of membranes in DNA transactions and discuss the potential role of Hfq-mediated regulation of these processes in *Escherichia coli*, where the protein is the best characterized. Special attention is given to the affinity of this small protein for both DNA and membranes, which might help explain some of the findings from recent experiments.

## 1. Introduction

**Figure 1:**
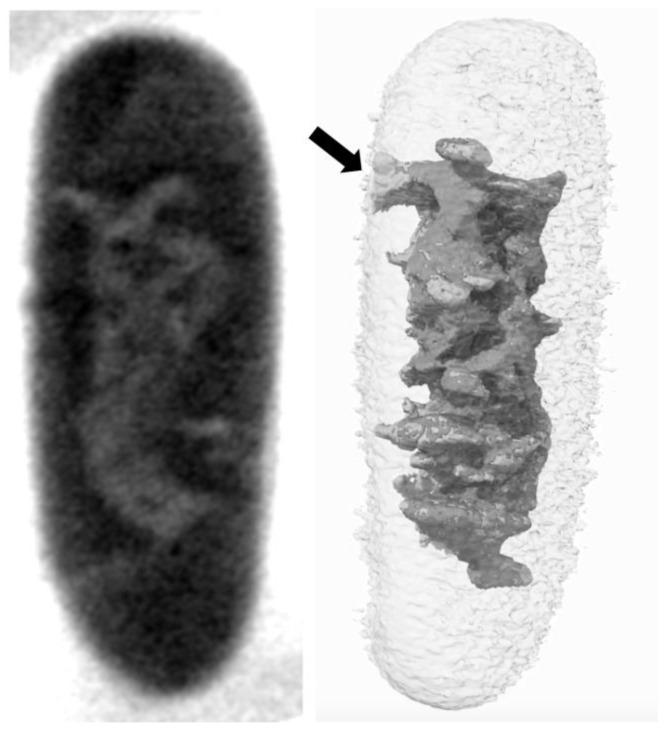
Native, unlabeled *E. coli* analyzed using Cryo-Soft X-ray Tomography (Cryo-SXT). **Left**: Tomographic slice reconstruction of a representative MG1655 cell in the exponential growth phase. The slice was extracted from absorbance-reconstructed volumes of the bacterium. **Right**: Visualization of the nucleoid volume for the same bacterium following segmentation. The interaction between the bacterial membrane and the nucleoid (indicated by the black arrow) is clearly visible due to the high imaging resolution [9]. Note that earlier reports using electron micrographs of *E. coli* showed the nucleoid in contact with the cell membrane [10], but these were later determined to be artifacts caused by cell fixation [11,12]. Here, cryo-fixation is used to avoid such artifacts [13].

The circular *Escherichia coli* genome of ~4.6 × 10^6^ bp, with a linear length of ~1.5 mm, must fit into a bacterial cell that is about 1–2 µm in length with a radius of ~0.5 µm. In spite of the necessary ~1000-fold compaction, DNA needs to be operational in supporting regulated gene expression; DNA replication leads to chromosome duplication and subsequent segregation into two daughter cells; repair of spontaneous, chemical, or physical DNA damage; and genetic recombination, when needed [1]. To complicate matters, a cell under active growth conditions may have multiple copies of the chromosome in various stages of replication. To facilitate the compaction of the chromosome, the DNA is bathed in counter ions, associated with many nucleoid-associated proteins (NAPs), negatively supercoiled, and folded into independent topological domains into a structure termed the nucleoid [2,3,4,5,6]. The nucleoid comprises the bacterial chromosome and, while it is safely ensconced within the bacterial cell, early studies reported its attachment to the bacterial membrane [3,7]. In recent years, less disruptive and noninvasive technologies revealed that connection to the bacterial membrane is less certain [5,6], although many clear examples of chromosome–membrane interactions exist [8] (Figure 1). A complete understanding of its structural and functional organization remains to be fully understood even after 50 years of research. As described below, evidence exists for functional and structural interactions between the membrane and bacterial chromosome. The myriad NAPs that coordinate and direct DNA transactions include those that interact with membranes.

Bacterial DNA metabolism involves a variety of processes crucial for maintaining genetic material transmission, its partition in daughter cells, and genome integrity. These processes are usually tightly regulated and often interact with bacterial cell membranes, allowing DNA-related machinery to be well-positioned for efficient function. For instance, some bacterial proteins involved in DNA replication are membrane-anchored. These physical interactions of DNA with membranes may occur directly or through DNA-binding proteins that have membrane affinity. As an example, *E. coli* DnaA, which activates the initiation of bacterial DNA replication, interacts with cardiolipin, a lipid present in the *E. coli* membrane [14]. Alternatively, FtsK, which plays a key role in chromosome segregation, physically interacts with the membrane through a membrane-spanning domain [15].

Recently, a new player associated with the bacterial membrane has emerged in the field of DNA metabolism [16]. This is the protein Hfq (Host factor Q) [17], a highly conserved RNA-binding protein that plays a crucial role in RNA-based regulation [18]. Hfq homologs are present in approximately 50% of bacterial species [19,20], with the majority of research focusing on Gram-negative and, in particular, *E. coli* Hfq. However, the role of Hfq in Gram-positive bacteria seems to differ significantly from that in Gram-negative species [21]. This review thus mainly focuses on *E. coli* Hfq. The main role of Hfq in Gram-negative bacteria is to stimulate base-pairing between small regulatory RNA (sRNA) and its target mRNA. As an RNA/RNA mediator, Hfq helps to regulate gene expression by altering mRNA stability and/or translation, with important consequences in stress responses (temperature, oxidative stress, nutrient deprivation, …) or in the modulation of biofilm formation or in virulence [22,23]. Nevertheless, the multiple roles of this protein are not limited to RNA-related functions, as it has been linked to a number of processes beyond RNA regulation and DNA shaping as an NAP. The biological roles of Hfq, identified from *hfq*-deficient mutants with very pleiotropic effects on cells, are many [24]. While facilitating RNA/RNA interactions, Hfq also binds to single- and double-stranded DNAs [25,26,27] and about 10–20% of the total protein is found to be associated with the bacterial chromosome, giving an average concentration of ~10–15 µM in the nucleoid [28]. Other subcellular locations of Hfq include 50% of the protein in close proximity to the membrane and the remaining 30% in the cytoplasm [28]. Hfq interacts with many proteins [29] and may well provide a bridge, connecting and orchestrating interactions between the bacterial chromosome and membrane.

**Figure 2:**
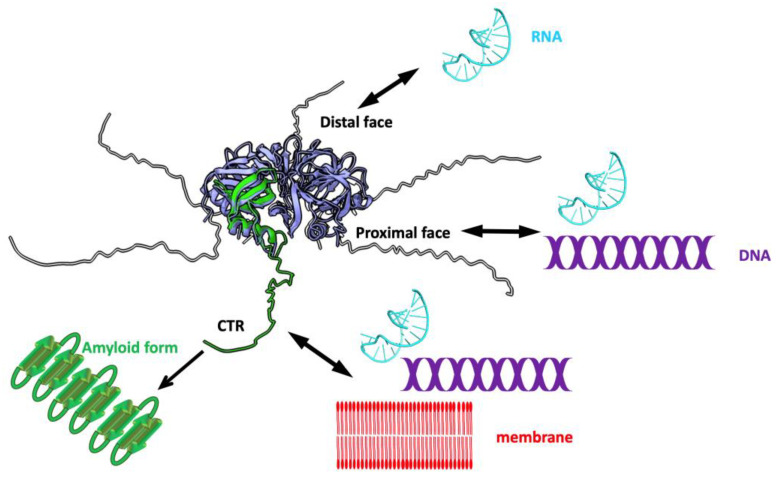
Molecular representation of full-length *E. coli* Hfq and associated functions. The NTR hexameric torus is represented in light blue with one monomer highlighted in green (PDB ID 3QHS). The structure of intrinsically disordered CTR is predicted by Alphafold (AF-P0A6X3-F1). To date, the CTR has not been visible in any experimental high-resolution structures. Both surfaces of the torus bind RNA but with different affinities. The proximal face, on which the α-helix is exposed, interacts with A-rich RNA sequences; the opposite face, named the distal face, binds uridine-rich RNAs [31,32]. The proximal surface is also involved in DNA binding [34]. Six CTRs emerge from the torus that interact with RNA (cyan), DNA (purple), and the membrane (red) [27,40,43]. The CTRs can also adopt an amyloid structure under specific conditions [38].

Structurally, *E. coli* Hfq is composed of two regions: a N-terminal region (NTR) that forms a hexameric toroidal structure with two well-differentiated faces, a distal and a proximal one (on which the α-helix is exposed) [30]. The distal and the proximal faces of the protein are both involved in the binding of nucleic acids with different specificities [31,32,33,34,35]. DNA molecules bind across the proximal face of the torus [34] (Figure 2). The RNA annealing function of Hfq mainly arises from this NTR region [30,32,36]. In addition to this NTR, six C-terminal regions (CTRs, 38 amino acid residues) extend outward from the central NTR core [30,31,37] (Figure 2). Until recently, the role and structure of its longer CTR were not well understood. However, recent studies have shown that the CTR adopts an amyloid-like structure [38,39]. Beyond its well-established functions in RNA- and DNA-related processes, Hfq also interacts with bacterial inner and outer membranes [40,41,42]. This association, initially thought to be relatively weak and reversible, with the protein lying on the membrane surface, has been revisited [40]. Indeed, Hfq can be inserted into the membrane using its amyloid C-terminal region [41,42]. Such interaction may thus be particularly significant for understanding DNA anchoring in the membrane.

This review highlights the multifaceted roles of Hfq in bacterial DNA physiology, extending beyond RNA regulation, in relation to Hfq’s interaction with the membrane. In fact, Hfq appears to be involved in the coupled transcription, translation, and insertion of nascent proteins into the membrane [44]. If we add the involvement of Hfq in DNA transactions that are also coupled to cell membranes, a complex picture of interactions appears, which is still partially unknown. Hence, understanding these interactions appears essential for comprehending the full spectrum of Hfq’s functions in bacterial cells.

## 2. Materials and Methods

This is a narrative review, based on the literature data from publications in English, recorded in the PubMed database (https://pubmed.ncbi.nlm.nih.gov/; last accessed on 21 February 2025). For the literature search, the following term was used: “Hfq and membrane”. The number of records found in this search was 163. Among them, 29 papers concerned DNA replication; 6 papers concerned other DNA transaction processes, like genetic recombination, DNA damage/mutagenesis, and repair; 2 papers concerned the outer membrane vesicle (OMV) DNA cargo-loading; 5 papers concerned the transposition; and 5 papers concerned the organization of the chromosome. Non-English articles were excluded (with one exception, describing the original proposal of the replicon model, published in French), as were those that did not address the problems of DNA transactions and membranes or Hfq directly. After such a selection, 47 articles were analyzed in detail. Other articles cited in this review are papers describing the properties of the Hfq protein, DNA–membrane interactions, and other issues related to the subject of this work.

## 3. Mechanisms of DNA-Mediated Membrane Interaction

Understanding the mechanisms of DNA–membrane interaction is fundamental for apprehending various biological events, including gene regulation, signal transduction, and membrane-associated protein functions. For this reason, it represents a significant area of study in biophysics and nanotechnology. The functional interactions and methods used to study them are summarized in the following paragraphs.

### 3.1. Direct Versus Indirect Membrane–DNA Interaction

DNA–membrane adhesion can be mediated through various interactions. In the absence of proteins, the *E. coli* surface displays a negative charge, which originates from the negatively charged lipids and lipopolysaccharide (LPS) molecules present in the Gram-negative outer membrane [45]. Anionic lipids in bacterial membranes include phosphatidylethanolamine (PE), phosphatidylglycerol (PG), and cardiolipin (CL) [45,46]. Cationic lipids are not naturally present in the *E. coli* membrane. Thus, the interaction between the *E. coli* membrane and DNA is not inherently favorable. Nevertheless, the negative lipids can be complexed with DNA via interactions with cations, such as calcium (Ca^2^⁺), magnesium (Mg^2^⁺), or sodium (Na⁺), which help neutralize the negative charges of both the membrane and DNA. This charge neutralization reduces the repulsive forces and allows DNA to interact with the membrane. This interaction has been shown to successfully mediate DNA adhesion with the *E. coli* lipid bilayer and is particularly important in the transformation process, where bacteria take up external DNA [47]. However, the most important factor that allows the membrane to interact with DNA is the presence of specific proteins.

Gram-negative bacteria, such as *E. coli*, have DNA-binding proteins that are either permanently or transiently membrane-bound. Membrane-inserted proteins, for instance, play a role in translocating DNA through the membrane barrier. As an example, in *E. coli*, proteins like TonB or FepA can assist in DNA uptake [48,49]. Other proteins, such as the nucleoid occlusion protein Noc, bind directly to DNA and associate with the cell membrane via an amphipathic helix without crossing the membrane [50]. Finally, an example of a protein that transiently interacts with membranes during replication initiation is DnaC in *E. coli* [51]. Together with the action of cations, these proteins enable the bacterial membrane to interact with DNA.

### 3.2. Methods to Analyze Membrane–DNA Interaction

The methods employed to study these interactions include *in vivo* techniques such as (i) the membrane Two-Hybrid Assay [52]; (ii) *in vivo* DNA footprinting to identify specific DNA regions bound by a membrane protein [53]; (iii) Chromatin Immunoprecipitation (ChIP) assays to detect DNA–membrane protein interactions within living cells [54]; and (iv) high-resolution *in vivo* cell imaging, such as cryo-EM or cryo-SXT, to obtain a detailed 3D visualization of bacterial ultrastructure. Cryo-EM enables high-resolution imaging of membranes, while cryo-SXT facilitates visualization of the nucleoid [12,13]. These *in vivo* analyses can be confirmed and analyzed in greater detail using *in vitro* methods. First, the direct interaction of the protein with liposomes can be assessed using various methods, such as ultracentrifugation, as shown in the case of DnaA, which plays a crucial role in the initiation of *E. coli* chromosome replication [51]. Molecular imaging techniques, such as Atomic Force Microscopy (AFM), can also be used [41,55,56]. Finally, Oriented Circular Dichroism (OCD) or polarized infrared spectroscopy can be used to study DNA interaction and/or insertion into the membrane [57]. A combination of AFM, FTIR, and OCD may allow discrimination of whether a protein involved in DNA membrane anchoring is peripheral or integral.

## 4. Regulatory Processes Related to the Importance of Membranes in Different DNA Transactions and the Potential Role of Hfq

As indicated in the previous section, interactions of DNA with bacterial membranes, either direct or indirect (mediated through DNA-binding proteins that have affinity to these structures) appear evident. In this section, we will focus on the membrane-related regulation of different biological processes based on DNA transactions.

### 4.1. DNA Replication

Replication of DNA is a fundamental process essential for all bacterial species. Duplication of the genetic material before the division of a cell is crucial to maintaining the integrity of the genome throughout generations. Thus, DNA replication events must be very precisely regulated. In the replicon model first proposed by Jacob and Brenner in 1963 [58], there is a specific site in DNA where the replication process starts, called the replication *origin* (abbreviated as *ori*). Replication initiation at *ori* is controlled by a factor, usually a protein, acting *in trans*. Subsequent studies indicated that this model is valid for both bacterial chromosomes and extrachromosomal replicating genetic elements (e.g., plasmids or bacteriophage genomes). However, a major question is how the timing and frequency of the initiation of DNA replication is regulated, as this process should determine the efficacy of duplication of the genetic material.

Even early studies suggested that the DNA replication initiation process might require the attachment of DNA to the bacterial membrane. For example, in one classical model, the bacteriophage λ genome, the phage DNA was attached to the membrane only under conditions supporting the replication process in *E. coli* [59]. Similar conclusions were made on the basis of studies of chromosome and plasmid DNA replication in *Bacillus subtilis* cells, where DNA–membrane associations appeared critical for DNA replication initiation [60]. In fact, the concept of membrane participation in the regulation of DNA duplication was a basis for a proposal on how to coordinate replication and partition [61,62]. This hypothesis was subsequently confirmed experimentally [63].

One obvious mechanism by which the association of DNA with membranes might control the replication initiation event is through the recruitment of crucial proteins required for DNA synthesis to a site on the membrane [64]. Proteins that reveal an affinity to both DNA and membrane, like the *B. subtilis* Noc protein, may play important roles in such reactions [50]. The binding of CTP to Noc regulates its binding to the membrane, which influences the cell division process [65]. Moreover, some membrane-associated proteins, like *E. coli* Bam proteins, significantly modulate the control of DNA replication [66]. Nevertheless, the importance of membranes for the regulation of DNA replication is not restricted to the assembly functions. Indeed, the activity of the replication initiator protein, called DnaA, which binds specifically to the bacterial chromosome replication origin sequence, called *oriC*, can be modulated by acidic phospholipids occurring in bacterial membranes. Early work demonstrated that DnaA can be found in the membrane [67], and more detailed experiments indicated that about 10% of the total pool of cellular DnaA is associated with membranes *in vivo* [14,51,68,69,70,71,72]. Importantly, membrane attachment activates DnaA for exchange in nucleotides (ADP *vs.* ATP) that this protein binds [70,72]. Particularly, phospholipids stimulate the conversion of the inactive form of DnaA, bound to ADP, to the form that positively regulates replication, bound to ATP. On the other hand, these compounds negatively regulate the DNA-binding activity of DnaA [8,73,74,75,76,77]. Interestingly, differences in levels of membrane acidic phospholipids influence DnaA activity significantly, thus affecting DNA replication. In addition, the expression of genes coding for enzymes involved in the metabolism of such lipids can be modulated by small regulatory RNAs (sRNAs) [78]. This points to the possible importance of factors controlling sRNA activities in the regulation of DNA replication.

Hfq also appears as a new player in the control of DNA replication [79]. Beyond its function in RNA/RNA annealing in plasmid replication [79], Hfq may indeed play various roles in the replication processes. First, Hfq, as a nucleoid-associated protein (NAP), may compact DNA locally and influence the shaping of DNA regions important for plasmid/chromosome segregation [80,81]. This could be particularly the case in the region of interaction with the membrane where the protein can anchor DNA [40].

Hfq, possessing both RNA- and DNA-binding activities, can differentially affect the replication of various replicons. For example, Hfq may significantly influence the control of replication of plasmids belonging to the ColE1-type group but not those derived from bacteriophage λ [16]. The simplest explanation for such differences might be that the mechanisms controlling replication initiation of ColE1-type replicons require RNA–RNA interactions, which could be affected by Hfq, while this type of regulation does not occur in bacteriophage λ. However, the full story may not be so simple. One possible mechanism by which Hfq could influence ColE1-type plasmid replication is its interplay with the Rom protein. This protein is a negative regulator of replication initiation that acts by enhancing the interactions between pre-primer RNA II and inhibitor RNA I. In fact, the effects of mutations in the *hfq* gene on the abundance of ColE1-type plasmids depend on the presence or absence of the functional *rom* gene [82]. A recent study demonstrated that Hfq can bind RNA I, and this interaction protects the negative regulator of ColE1 replication against degradation by RNase E [83]. Thus, elevated levels of RNA I can cause more effective inhibition of ColE1 replication initiation.

Another activity of Hfq, and especially its C-terminal domain, was demonstrated to affect the DNA replication process significantly. Specifically, a lack of this domain resulted in less effective replication of the M13 bacteriophage, which has a ssDNA genome [26]. Thus, Hfq-mediated ssDNA-binding could be required at some stages of M13 DNA replication, which contains single-stranded intermediates.

Hfq also influences replication and chromosome duplication of the bacterial chromosome by regulating the actions of small regulatory RNAs. An example of such RNA is DsrA, which is involved in the control of DNA replication initiation from *oriC* in *E. coli*, whose action is Hfq-dependent [84]. However, the most obvious connection of Hfq activity to the regulation of *E. coli* chromosome replication is its role in the control of expression of the *dnaA* gene. Namely, an abundance of mRNA molecules derived from *dnaA* is negatively regulated by rnTrpL, which is a sRNA, facilitating degradation of this transcript encoding the replication initiation protein DnaA. This negative regulation by rnTrpL requires a Hfq function [85]. Hence, by influencing levels of DnaA, the Hfq protein is evidently involved in the regulation of bacterial chromosome replication. The effects of this protein on DNA topology might enhance these effects. In turn, interactions of DnaA with membranes bring the connection between replication, membranes, and Hfq even closer. Detailed studies have identified domains of the DnaA replication initiator protein responsible for the link between membranes and DNA replication regulation, mainly via cardiolipin microdomains [86]. A deletion within the linker domain (of previously unknown function) of DnaA resulted in a deficiency in the association of this protein with anionic membrane vesicles [87]. As both Hfq and DnaA proteins concentrate on cardiolipin microdomains in the membrane [14,88,89], they could physically interact and coordinate to regulate replication. Note that the role of DnaA protein acetylation remains poorly understood; nevertheless, Hfq has been shown to interact with an acetyltransferase and could influence DnaA post-translational modification (PTM) [90,91].

Another interesting aspect of DNA replication regulation is the effects of epigenetic modifications. DNA methylation plays a crucial role in this, as only hemi-methylated DNA (in which only one DNA strand is methylated) can be efficiently bound to membranes [92]. Since this specific binding is mediated by the negative regulator of *E. coli* replication initiation, the SeqA protein [93], a regulatory role for DNA–membrane interactions appears obvious. Increased methylation leads to a stronger binding affinity of Hfq, slowing its diffusion along DNA [94]. Interestingly, truncated versions of Hfq, lacking the CTR domain, display reduced DNA binding affinity and shorter residence times, resulting in less efficient DNA compaction [80,94]. This control of Hfq mobility and binding by DNA methylation may, therefore, have potential implications for replication.

Interestingly, DnaA and SeqA proteins influence the properties of cell membranes, as demonstrated by an increased permeability of membranes and a higher sensitivity to ethanol and deoxycholate of *dnaA* and *seqA* mutants [95]. Conversely, Hfq seems to increase membrane permeability [41]. In addition to its role in sRNA-based regulation, Hfq may directly create pores in the membranes and may also influence membrane permeability indirectly by modulating lipid composition and/or pH homeostasis [41,96].

Finally, the major replicative DNA polymerase (DNAP), DNA polymerase III, also interacts with the membrane [97]. The membrane may provide a stable environment necessary to prevent the disassembly of the replication machinery during the elongation phase of DNA replication. While there is no substantial evidence suggesting a direct interaction between Hfq and DNA polymerase in terms of physical binding, Hfq could still influence the function of DNA polymerase by regulating factors that impact DNA replication, such as its local compaction. Additionally, Hfq could modulate the expression of genes that code for DNA polymerase or other proteins involved in the replication machinery through its interaction with regulatory RNAs.

Although a direct interaction between DNA polymerase and Hfq has not been proven, RNA polymerase, conversely, physically interacts with the RNA chaperone [98]. Bacterial RNA polymerase is primarily found in the cytoplasm, specifically in the nucleoid region of the cell. Unlike in eukaryotic cells, where RNA polymerase can be associated with the nuclear envelope, bacterial RNA polymerase does not typically reside at the membrane. However, in some contexts, RNA polymerase might be involved in membrane-related processes. This includes the synthesis of membrane proteins or components involved in stress adaptation, allowing the translational machinery to be positioned close to the membrane for efficient protein insertion and/or localization using the Signal Recognition Particle (SRP) [99]. This coupled transcription, translation, and insertion of nascent proteins into the membrane is referred to as membrane transertion [44,100,101]. Notably, a physical link between Hfq, RNA polymerase (RNAP), and the ribosomal protein S1 has been reported [98]. Additionally, there is evidence suggesting that Hfq can act as a scaffold that brings together different RNA molecules, RNA polymerase, and other transcriptional regulators in a complex that could localize near the membrane in specific cases [102,103]. Acetylation of S1 has also been described, and Hfq could influence this process [91,104]. Therefore, Hfq is undoubtedly involved in transertion, highlighting its importance in membrane-associated processes.

GyrA, which encodes the A subunit of DNA gyrase (topoisomerase II), also interacts with the SRP protein Ffh to insert nascent membrane proteins into the membrane. This interaction suggests a potential link between GyrA, which interacts with Hfq [29], and membrane-associated processes. The DNA-induced negative supercoiling of bacterial DNA by gyrase is necessary for replication [105]. While these findings indicate that GyrA may interact with membrane components or influence membrane-related processes, direct interactions between GyrA and the bacterial membrane have not been established. Even though the detailed molecular mechanism and possible direct interaction between Hfq and GyrA are not fully understood, they could represent a sophisticated network of gene regulation, with Hfq playing an indirect role in modulating the expression of genes involved in DNA supercoiling [81].

In addition to replication, another putative role for Hfq in bacterial cell division is its involvement in MreB regulation. MreB is an actin-like protein that plays a central role in cell wall biosynthesis required to maintain bacterial cell shape. Additionally, MreB influences plasmid partitioning by organizing the division machinery and is implicated in co-replicational DNA segregation [106]. Interestingly, MreB’s expression is controlled by sRNA and Hfq [107], and the proteins possibly co-localize in the cell, both forming a cytoskeletal-like organization near the inner membrane [108]. A transient interaction between Hfq and MreB may help coordinate replication and cell division.

In summary, the interplay between DNA, replication proteins, and membranes is not only of structural importance but it is also involved in the precise regulation of the replication initiation frequency. Activities of both positive (e.g., DnaA) and negative (e.g., SeqA) regulators are involved in DNA–membrane interactions, influencing the membrane properties but also being influenced by lipid and protein components of these membranes. Therefore, there is a complex structural and functional network, composed of DNA, proteins, and membranes, operating especially in the *ori* region of the replicon, ensuring precise control of the replication initiation event Hfq is evidently involved in the regulatory processes related to the control of DNA replication, partially due to the documented interactions with cell membranes. Still, many details of the mechanisms regulating this process remain to be elucidated.

### 4.2. Genetic Recombination, DNA Damage/Mutagenesis and Repair

Reports on the involvement of membranes in DNA recombination, mutagenesis, and repair are definitely scarcer than those on DNA replication. Nevertheless, cell membranes undoubtedly take part in these processes. A crucial finding is that the RecA protein, which participates in both genetic recombination and the stress response to DNA damage (including DNA repair processes), is associated with cell membranes [109]. Importantly, this property is characteristic only for the activated form of RecA, implying that the bacterial SOS response might be regulated through RecA–membrane interactions [109]. Such interactions were also found in complexes containing DNA during the bacterial transformation process, indicating that the triple complexes might be important in RecA-mediated DNA transactions [110]. It must be noted that Hfq interacts with RecA and that it could thus influence genetic recombination [29]. Experimental demonstration of the role of Hfq in homologous recombination strengthened this possibility [26]. Indeed, the protein induces significant structural changes and alters the helical parameters of single-stranded DNA, particularly through its C-terminal amyloid-like domain, providing new insights into the regulation of DNA recombination, possibly in the vicinity of the inner membrane. The physiological relevance of this interaction was demonstrated using a λ recombination assay that was impacted in the Δ*hfq* mutant [26].

When DNA damage occurs, a specific replication checkpoint is necessary to delay cell division until the genetic material is repaired. The mechanism of DNA damage checkpoint involves membrane-bound cell division inhibitors, coupling the repair/recombination machinery to cell division through a membrane [111]. Interestingly, in some bacteria, such a mechanism linking the DNA damage response and cell cycle does not exist, although the cells can still survive and repair their genetic material [112]. One should note that the detection of DNA damage is performed by specific proteins on the basis of detecting changes in DNA structure. Since Hfq may influence DNA topology, one might speculate that this protein might also be involved in the replication checkpoint recognition.

*Deinococcus radiodurans* is an especially interesting model in studies on DNA damage and repair as this bacterium is especially resistant to the actions of DNA-damaging agents, particularly radiation. A protein kinase, DR2518, that is involved in the signaling process during the DNA damage response plays a crucial role in both resistance to radiation and repair of DNA strand breaks, and it is a membrane-associated protein [113]. This discovery provided additional evidence for the coupling of the cell membrane to DNA repair. In this light, it is worth noting that stabilization of the cell membrane during dehydration of *D. radiodurans* was suggested to be connected to the DNA repair process, mediated by RecA and PprA proteins [114]. Nevertheless, the Hfq regulon that contributes to nucleoid packaging in *E. coli* is missing in *D. radiodurans*.

In conclusion, the processes of DNA recombination and repair require cell membranes. However, the exact mechanisms of the involvement of membranes in these DNA transactions are still relatively poorly understood. Thus, finding newly identified factors that might contribute to the control of these processes is perhaps required to learn more about these complicated yet essential cellular functions. Indicating the importance of Hfq in genetic recombination is an example of such discoveries which added to our understanding of the mechanisms of the exchange of DNA fragments between homologous molecules.

### 4.3. Other DNA Transactions: Transposition, OMV DNA Cargo-Loading, Nucleoid Organization

Besides the roles mentioned in replication, Hfq plays other roles in DNA transactions. One of these roles is to load DNA as cargo in outer membrane vesicles (OMVs) [115]. OMVs are small, spherical structures naturally released from the outer membrane of Gram-negative bacteria [116]. They contain various components, including lipids, proteins, and nucleic acids, such as DNA or RNA. The presence of DNA in OMVs may be linked to processes such as horizontal gene transfer or bacterial communication. As Hfq seems to actively participate in OMV biogenesis and nucleic acid cargo loading, it may also play a critical role in this process [117].

The transposition of mobile genetics elements in bacterial cells is also influenced by the activities of the Hfq protein. The first demonstration of the involvement of Hfq in transposition was that this protein modulates Tn*10*/IS*10* transposition frequency by influencing the regulatory system consisting of RNA-IN and RNA-OUT molecules. Specifically, Hfq binds to both these RNAs, enhancing their interactions [118]. However, Hfq also inhibited Tn*10* transposition even if no antisense RNA was produced [119]. This ostensible paradox was solved by demonstrating that Hfq is able to bind directly to the ribosome-binding site of the mRNA coding for IS*10* transposase. Such binding inhibits mRNA translation, causing significantly lower levels of the enzyme responsible for the movement of the mobile genetic element [120]. The production of another transposase, which is encoded by Tn*5*, is also negatively regulated by Hfq. However, in this case, transcription of the gene encoding the transposase is inhibited [121]. These findings revealed that there are at least three modes of Hfq-mediated regulation of the transposition frequency: enhancement of RNA–RNA interactions, impairment of translation of the transposase mRNA, and repression of transcription of a transposase-encoding gene [122].

The bacterial chromosome in *E. coli* is organized into a three-dimensional nucleoid structure wherein the DNA is folded and organized with RNA and a myriad of nucleoid-associated proteins (see the following for a recent review [5,6]). At least twelve NAPs are recognized as being associated with the nucleoid [123,124], and specific nucleoid-associated RNAs (naRNAs) have also been identified [125,126]. Significantly, evidence abounds for the interaction of the DNA within the nucleoid with the bacterial membrane [3,7,8]. While Hfq is not typically identified as a protein involved in nucleoid structural organization, it nevertheless may be very important in the positioning of the nucleoid within the cell. While the nucleoid may be compartmentalized, in part, by liquid–liquid phase separation from other components of the cytoplasm [5], certain chromosomal loops active in transcription, or functional for replication and chromosome segregation, may well interact with membranes. Protein synthesis involves a physical continuum from DNA to RNA to protein and, at times, to membranes. As Hfq has functional transactions with all these entities [29], it might not be unexpected for it to contribute to the compartmental organization of the cell. In addition, under stress conditions, Hfq appears to undergo liquid–liquid phase separation (LLPS) to sequester sRNAs or RNaseE [127,128]. Therefore, Hfq may participate, both indirectly and directly, in the organization, compartmentalization, functions, and membrane interactions of the nucleoid with the *E. coli* cell.

## 5. Conclusions

**Figure 3:**
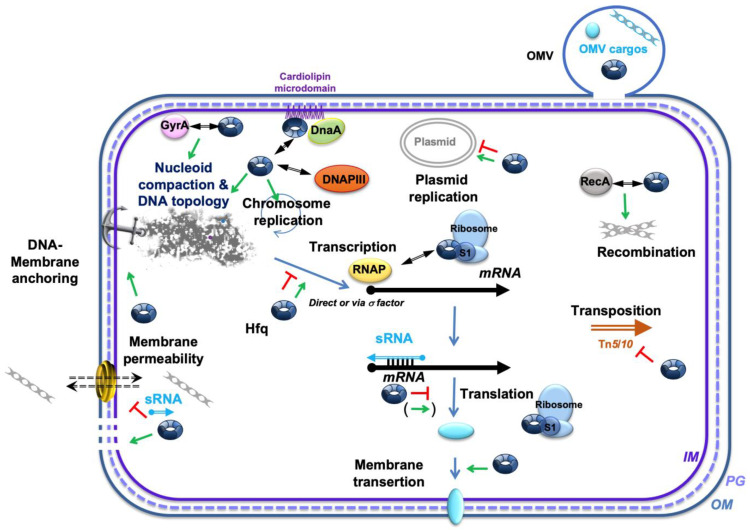
Network of main Hfq-dependent processes and regulatory interactions near the membrane. Hfq is implicated in several cellular processes related to both chromosomal and plasmid DNA replication, nucleoid anchoring at the membrane, and coordination of cell division. It also plays a role in nucleoid compaction, DNA transposition, transcription/translation, and membrane-protein transertion. Additionally, Hfq is believed to influence genetic recombination through its interaction with RecA, and Hfq interacts with the DNA gyrase subunit GyrA. Moreover, Hfq and DnaA may co-localize on cardiolipin microdomains, altering membrane permeability [41,129]. Hfq is also thought to impact the loading of DNA cargo into outer membrane vesicles (OMVs) and to influence membrane permeability. The sRNA regulators controlling mRNAs are shown as a thin blue arrow; Hfq is represented by a blue toroidal hexamer; mRNAs are depicted as thick black lines; the 5′ and 3′ ends of the mRNA are depicted by a “ball and arrowhead”, respectively; the positive and negative regulations are indicated by green arrows and red “T’s”, respectively; the double arrowhead symbolizes a (putative) physical interactions between proteins; the dotted line symbolizes peptidoglycan (PG) between the outer (OM) and inner (IM) membranes.

The simple bacterial model system, *E. coli*, reveals increasing complexity as the understanding of this organism deepens. This is evident in understanding DNA transactions including DNA replication, genetic recombination, transposition, DNA repair, and control of gene expression. Rather than these processes operating in isolation within the cytoplasm of a bacterial cell, it is becoming evident that many DNA transactions may be operating, at times, compartmentalized and organized in association with the bacterial membrane (Figure 3). The interactions of DNA with the membrane have been the subject of intensive investigation. The site for chromosomal DNA replication and the site where chromosomes are segregated into daughter cells are clearly associated with the membrane. While some DNA-centered and membrane-involving processes are relatively well-understood, the involvement with the membrane of other transactions, including recombination, transposition, horizontal gene transfer, OMV cargo loading, and gene expression, are less understood. Some processes may involve DNA–membrane contact and communication, but they are not sufficiently investigated to present a complete picture of their functional organization with membranes. The architectural organization of the bacterial chromosome has both structural and functional consequences, and its functional interactions with the bacterial membrane are beginning to be revealed. The dynamic nature of the chromosome and the membrane, including fluidity and changing permeability, as well as the transient nature of interactions between them, might contribute to the difficulty of understanding these interactions. Recent findings, summarized and discussed in this review, suggest that Hfq, a NAP interacting with DNA, RNA, and the membrane, could play important roles in these processes.

## Data Availability

No new data were created or analyzed in this study. Data sharing is not applicable to this article.

## Abbreviations

The following abbreviations are used in this manuscript:

sRNA	Small noncoding RNA
IM/OM	Inner/outer membrane
NAP	Nucleoid-Associated Protein
RNAP/DNAP	RNA or DNA polymerase
Tn	Transposon
OMV	Outer membrane vesicles
LLPS	Liquid–Liquid Phase Separation
PTM	Post-translational modification
SRP	Signal Recognition Particle
